# 1,4-Bis(4,5-dihydro-1*H*-imidazol-2-yl)benzene–4-amino­benzene­sulfonic acid–water (1/2/2)

**DOI:** 10.1107/S1600536809032504

**Published:** 2009-08-22

**Authors:** Shao-Ming Shang, Chun-Xia Ren, Xin Wang, Lu-De Lu, Xu-Jie Yang

**Affiliations:** a School of Chemical and Material Engineering, Nanjing University of Science and Technology, 200 Xiaolingwei Road, Nanjing, Jiangsu Province 210094, People’s Republic of China; b School of Chemical and Material Engineering, Jiangnan University, 1800 Lihu Road, Wuxi, Jiangsu Province 214122, People’s Republic of China

## Abstract

The asymmetric unit of the title compound, C_12_H_14_N_4_·2C_6_H_7_NO_3_S·2H_2_O, contains one half of a centrosymmetric 1,4-bis­(4,5-dihydro-1*H*-imidazol-2-yl)benzene (bib) molecule, one 4-amino­benzene­sulfonic acid molecule and one water mol­ecule. In the bib molecule, the imidazole ring adopts an envelope conformation. The benzene rings of bib and 4-aminobenzenesulfonic acid are oriented at a dihedral angle of 21.89 (4)°. In the crystal structure, inter­molecular N—H⋯O, O—H⋯N and O—H⋯O inter­actions link the mol­ecules into a three-dimensional network. Weak π–π contacts between the benzene and imidazole rings and between the benzene rings [centroid–centroid distances = 3.895 (1) and 3.833 (1) Å, respectively] may further stabilize the structure.

## Related literature

For general background, see: Jeffrey (1997 ▶); Thaimattam *et al.* (1998 ▶). For related structures, see: Ren *et al.* (2004*a* ▶,*b* ▶, 2007 ▶, 2009 ▶). For imidazole bond lengths, see: Haga *et al.* (1996 ▶); Hammes *et al.* (2005 ▶).
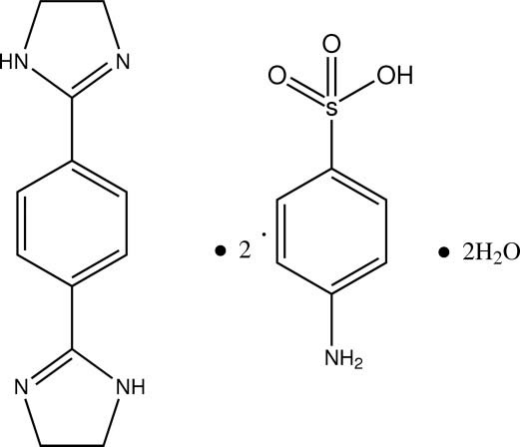


## Experimental

### 

#### Crystal data

C_12_H_14_N_4_·2C_6_H_7_NO_3_S·2H_2_O
*M* *_r_* = 596.70
Orthorhombic, 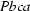

*a* = 13.6306 (11) Å
*b* = 12.698 (1) Å
*c* = 15.5907 (13) Å
*V* = 2698.5 (4) Å^3^
*Z* = 4
Mo *K*α radiation
μ = 0.26 mm^−1^
*T* = 273 K
0.15 × 0.12 × 0.10 mm

#### Data collection

Bruker SMART CCD area-detector diffractometer
Absorption correction: multi-scan (*SADABS*; Bruker, 1998 ▶) *T*_min_ = 0.962, *T*_max_ = 0.975
11859 measured reflections
2346 independent reflections
1856 reflections with *I* > 2σ(*I*)
*R*_int_ = 0.036

#### Refinement

*R*[*F* ^2^ > 2σ(*F* ^2^)] = 0.045
*wR*(*F* ^2^) = 0.140
*S* = 1.08
2346 reflections
182 parameters
All H-atom parameters refined
Δρ_max_ = 0.45 e Å^−3^
Δρ_min_ = −0.42 e Å^−3^

### 

Data collection: *SMART* (Bruker, 1998 ▶); cell refinement: *SAINT* (Bruker, 1998 ▶); data reduction: *SAINT*; program(s) used to solve structure: *SHELXTL* (Sheldrick, 2008 ▶); program(s) used to refine structure: *SHELXTL*; molecular graphics: *SHELXTL* and *PLATON* (Spek, 2009 ▶); software used to prepare material for publication: *SHELXTL* and *PLATON*.

## Supplementary Material

Crystal structure: contains datablocks global, I. DOI: 10.1107/S1600536809032504/hk2751sup1.cif

Structure factors: contains datablocks I. DOI: 10.1107/S1600536809032504/hk2751Isup2.hkl

Additional supplementary materials:  crystallographic information; 3D view; checkCIF report

## Figures and Tables

**Table 1:** Hydrogen-bond geometry (Å, °)

*D*—H⋯*A*	*D*—H	H⋯*A*	*D*⋯*A*	*D*—H⋯*A*
O3—H3*A*⋯O4	0.82	2.00	2.809 (3)	168
N1—H1*A*⋯O2^i^	0.86	2.22	2.960 (3)	145
N1—H1*B*⋯O3^ii^	0.86	2.43	3.200 (3)	150
N1—H1*B*⋯O1^ii^	0.86	2.46	3.170 (4)	141
N2—H1⋯O3^iii^	0.86	2.07	2.897 (3)	161
O4—H4*A*⋯N3^iv^	0.85	2.08	2.760 (3)	136
O4—H4*B*⋯O1^v^	0.85	2.20	2.817 (3)	130

Symmetry codes: (i) 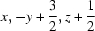; (ii) 
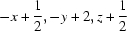
; (iii) 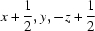; (iv) 
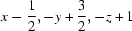
; (v) 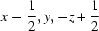.

**Fig. 1.**
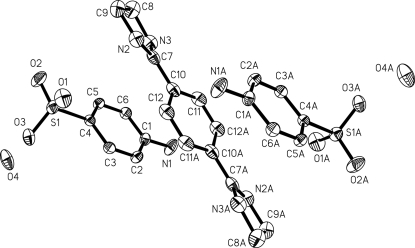
The molecular structure of the title molecule, with the atom-numbering scheme. Hydrogen bond is shown as dashed line [symmetry code ('): 1 - x, 2 - y, 1 - z].

**Fig. 2.**
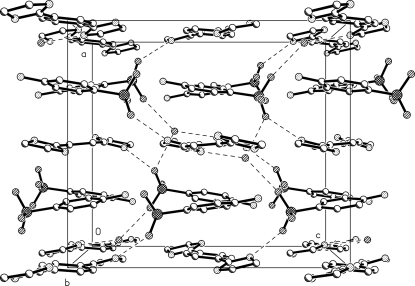
A partial packing diagram for the title compound. Hydrogen bonds are shown as dashed lines.

## supplementary crystallographic information

### Comment

Attention has recently focused on the use of supramolecular interactions such as hydrogen bonding and π–π interactions, in addition to coordinate bonds, in the controlled assembly of supramolecular architectures (Jeffrey, 1997). Hydrogen bonds often play a dominant role in crystal engineering because of their combine strength with directionality (Thaimattam *et al.*, 1998). On the other hand, supramolecular systems sustained by soft connections, such as hydrogen bonds, are comparatively more flexible and sensitive to the chemical environment. Consequently hydrogen-bond sustained systems are less designable and remain to be further investigated. We described previously a number of such metal complexes, including imidazole ligand, and have concluded that hydrogen bonding involving this group influences the geometry around the metal atom and the crystallization mechanism (Ren *et al.*, 2004a; Ren *et al.*, 2004b; Ren *et al.*, 2007; Ren *et al.*, 2009). We reported herein the synthesis and crystal structure of the title compound.

The asymmetric unit of the title compound contains one-half of 1,4-bis(4,5-di-hydro-1*H*-imidazol-2-yl)benzene (bib) ligand, one 4-aminobenzenesulfonic acid (SA) and one water molecules. In bib, the imidazole ring B (N2/N3/C7-C9) adopts envelope conformation with atom C8 displaced by -0.185 (3)Å from the plane of the other ring atoms. Rings A (C1-C6) and C (C10/C11/C12/C10'/C11'/C12') [symmetry code ('): 1 - x, 2 - y, 1 - z] are, of course, planar and they are oriented at a dihedral angle of 21.89 (4)°.

In the crystal structure, intramolecular O-H···O and intermolecular N-H···O, O-H···N and O-H···O interactions (Table 1) link the molecules into a three-dimensional network (Fig. 2), in which they may be effective in the stabilization of the structure. The π–π contacts between the benzene and imidazole rings and between the benzene rings, Cg1—Cg2 and Cg1—Cg3, [where Cg1, Cg2 and Cg3 are centroids of the rings A (C1-C6), B (N2/N3/C7-C9) and C (C10/C11/C12/C10'/C11'/C12'), respectively] may further stabilize the structure, with centroid-centroid distances of 3.895 (1) and 3.833 (1) Å, respectively.

### Experimental

For the preparation of 1,4-bis(4,5-dihydro-1*H*-imidazol-2-yl)benzene, (bib), 1,4-benzenedicarboxylic acid (2.31 g, 13.9 mmol), ethylenediamine (3.70 ml, 50 mmol), ethylenediamine dihydrochloride(6.64 g, 50 mmol) and toluene-*p*-sulfonic acid (0.208 g, 1.09 mmol) were added to the solvent of ethyleneglycol (20 ml), and the mixture was refluxed for 3 h. About half of the ethylene glycol solvent was then slowly removed by distillation. The residue was dissolved in a mixture of water (40 ml) and concentrated HCl (11 *M*, 3 ml). The addition of 50% aqueous NaOH gave a yellow precipitate that was purified by recrystallization. The ligand bib was obtained in 83% based on 1,4-benzenedicarboxylic acid (*ca* 2.50 g). Found: C 66.98; H 6.92; N 26.08%. Calc. for C12H14N4: C 67.27; H 6.59; N 26.15%. Main IR bonds (KBr, cm^-1^): 3188*m*, 2936*m*, 2866*m*, 1606 s, 1532 s, 1466 s, 1345*m*, 1270 s, 1191w, 1080w, 981*m*, 907w, 767w, 687*m*. For the preparation of the title compound, to a solution of bib (0.043 g, 0.2 mmol) in MeOH (15 ml), an aqueous solution (5 ml) of SA (0.068 g, 0.4 mmol) was added. The solution was allowed at room temperature in air for 3 d by slow evaporation. Large yellow prismatic crystals were obtained, which were collected by filtration, washed with water and dried in vacuum desiccator over silica gel (0.047 g, 54%). Main IR bonds (KBr,cm^-1^): 3424*m*, 3354*m*, 3249w, 1655w, 1603*m*, 1507*m*, 1119*m*, 1024 s, 1001*m*, 698*m*, 569w.

### Refinement

H atoms were positioned geometrically, with N-H = O.86 Å (for NH and NH_2_), O-H = 0.82 Å (for OH) and 0.85 Å (for H_2_O) and C-H = 0.93 and 0.97 Å for aromatic and methylene H, respectively, and constrained to ride on their parent atoms, with U_iso_(H) = xU_eq_(C,N,O), where x = 1.5 for OH H and x = 1.2 for all other H atoms.

### Crystal data

**Table:**

C_12_H_14_N_4_·2C_6_H_7_NO_3_S·2H_2_O	*F*(000) = 1256
*M**_r_* = 596.70	*D*_x_ = 1.469 Mg m^−^^3^
Orthorhombic, *P**b**c**a*	Mo *K*α radiation, λ = 0.71073 Å
Hall symbol: -P 2ac 2ab	Cell parameters from 3721 reflections
*a* = 13.6306 (11) Å	θ = 2.6–26.3°
*b* = 12.698 (1) Å	µ = 0.26 mm^−^^1^
*c* = 15.5907 (13) Å	*T* = 273 K
*V* = 2698.5 (4) Å^3^	Block, yellow
*Z* = 4	0.15 × 0.12 × 0.10 mm

### Data collection

**Table:**

Bruker SMART CCD area-detector diffractometer	2346 independent reflections
Radiation source: fine-focus sealed tube	1856 reflections with *I* > 2σ(*I*)
graphite	*R*_int_ = 0.036
φ and ω scans	θ_max_ = 25.1°, θ_min_ = 2.6°
Absorption correction: multi-scan (*SADABS*; Bruker, 1998)	*h* = −16→15
*T*_min_ = 0.962, *T*_max_ = 0.975	*k* = −15→15
11859 measured reflections	*l* = −18→14

### Refinement

**Table:**

Refinement on *F*^2^	Secondary atom site location: difference Fourier map
Least-squares matrix: full	Hydrogen site location: inferred from neighbouring sites
*R*[*F*^2^ > 2σ(*F*^2^)] = 0.045	All H-atom parameters refined
*wR*(*F*^2^) = 0.140	*w* = 1/[σ^2^(*F*_o_^2^) + (0.0724*P*)^2^ + 1.4974*P*] where *P* = (*F*_o_^2^ + 2*F*_c_^2^)/3
*S* = 1.08	(Δ/σ)_max_ < 0.001
2346 reflections	Δρ_max_ = 0.45 e Å^−^^3^
182 parameters	Δρ_min_ = −0.42 e Å^−^^3^
0 restraints	Extinction correction: *SHELXTL* (Sheldrick, 2008), Fc^*^=kFc[1+0.001xFc^2^λ^3^/sin(2θ)]^-1/4^
Primary atom site location: structure-invariant direct methods	Extinction coefficient: 0.0010 (5)

### Special details

**Table:**

Geometry. All e.s.d.'s (except the e.s.d. in the dihedral angle between two l.s. planes) are estimated using the full covariance matrix. The cell e.s.d.'s are taken into account individually in the estimation of e.s.d.'s in distances, angles and torsion angles; correlations between e.s.d.'s in cell parameters are only used when they are defined by crystal symmetry. An approximate (isotropic) treatment of cell e.s.d.'s is used for estimating e.s.d.'s involving l.s. planes.
Refinement. Refinement of *F*^2^ against ALL reflections. The weighted *R*-factor *wR* and goodness of fit *S* are based on *F*^2^, conventional *R*-factors *R* are based on *F*, with *F* set to zero for negative *F*^2^. The threshold expression of *F*^2^ > σ(*F*^2^) is used only for calculating *R*-factors(gt) *etc*. and is not relevant to the choice of reflections for refinement. *R*-factors based on *F*^2^ are statistically about twice as large as those based on *F*, and *R*- factors based on ALL data will be even larger.

### Fractional atomic coordinates and isotropic or equivalent isotropic displacement parameters (Å^2^)

**Table:**

	*x*	*y*	*z*	*U*_iso_*/*U*_eq_	
S1	0.20619 (5)	0.82812 (5)	0.31074 (4)	0.0423 (3)	
O1	0.29197 (15)	0.8625 (2)	0.26382 (13)	0.0688 (7)	
O2	0.1875 (2)	0.71668 (16)	0.30647 (13)	0.0681 (7)	
O3	0.12151 (14)	0.88982 (15)	0.28322 (11)	0.0536 (5)	
H3A	0.0729	0.8714	0.3104	0.080*	
O4	−0.06007 (17)	0.8498 (2)	0.36193 (13)	0.0774 (8)	
H4A	−0.0646	0.8028	0.4006	0.093*	
H4B	−0.1105	0.8846	0.3470	0.093*	
N1	0.2770 (2)	0.9242 (2)	0.67696 (14)	0.0615 (8)	
H1A	0.2775	0.8740	0.7140	0.074*	
H1B	0.2884	0.9878	0.6930	0.074*	
N2	0.51620 (17)	0.7721 (2)	0.34661 (15)	0.0518 (6)	
H1	0.5368	0.8180	0.3103	0.062*	
N3	0.46812 (18)	0.70826 (19)	0.46926 (16)	0.0530 (6)	
C1	0.25816 (19)	0.90302 (19)	0.59303 (15)	0.0384 (6)	
C2	0.2594 (2)	0.98296 (19)	0.53090 (16)	0.0398 (6)	
H2	0.2705	1.0522	0.5476	0.048*	
C3	0.24456 (19)	0.96036 (18)	0.44548 (15)	0.0368 (6)	
H3	0.2470	1.0141	0.4050	0.044*	
C4	0.22603 (17)	0.85807 (18)	0.41959 (15)	0.0327 (5)	
C5	0.22330 (18)	0.77832 (18)	0.48013 (15)	0.0362 (6)	
H5	0.2105	0.7095	0.4631	0.043*	
C6	0.2394 (2)	0.80063 (19)	0.56551 (15)	0.0392 (6)	
H6	0.2378	0.7463	0.6055	0.047*	
C7	0.49343 (18)	0.7937 (2)	0.42682 (17)	0.0441 (7)	
C8	0.4824 (3)	0.6141 (3)	0.4154 (2)	0.0657 (9)	
H8A	0.4241	0.5703	0.4149	0.079*	
H8B	0.5377	0.5726	0.4351	0.079*	
C9	0.5021 (3)	0.6610 (3)	0.3270 (2)	0.0604 (8)	
H9A	0.5604	0.6307	0.3013	0.073*	
H9B	0.4468	0.6506	0.2889	0.073*	
C10	0.49700 (19)	0.8996 (2)	0.46426 (16)	0.0446 (7)	
C11	0.5064 (2)	0.9132 (2)	0.55335 (17)	0.0513 (7)	
H11	0.5105	0.8546	0.5890	0.062*	
C12	0.4905 (2)	0.9878 (2)	0.41216 (17)	0.0514 (7)	
H12	0.4839	0.9796	0.3532	0.062*	

### Atomic displacement parameters (Å^2^)

**Table:**

	*U*^11^	*U*^22^	*U*^33^	*U*^12^	*U*^13^	*U*^23^
S1	0.0514 (5)	0.0491 (4)	0.0264 (4)	−0.0001 (3)	0.0007 (3)	−0.0023 (3)
O1	0.0604 (14)	0.1066 (17)	0.0396 (12)	−0.0006 (13)	0.0178 (10)	0.0025 (11)
O2	0.114 (2)	0.0497 (12)	0.0407 (12)	−0.0013 (12)	−0.0076 (11)	−0.0109 (9)
O3	0.0497 (12)	0.0712 (13)	0.0399 (10)	−0.0024 (10)	−0.0102 (9)	0.0053 (9)
O4	0.0590 (14)	0.128 (2)	0.0449 (13)	0.0017 (14)	−0.0103 (10)	0.0270 (13)
N1	0.097 (2)	0.0549 (15)	0.0321 (12)	−0.0210 (14)	−0.0048 (12)	−0.0035 (10)
N2	0.0504 (14)	0.0678 (16)	0.0371 (13)	−0.0018 (12)	0.0070 (11)	0.0087 (11)
N3	0.0438 (14)	0.0646 (15)	0.0507 (14)	−0.0019 (12)	0.0108 (11)	0.0103 (12)
C1	0.0396 (14)	0.0445 (13)	0.0312 (13)	−0.0052 (12)	0.0007 (11)	−0.0021 (10)
C2	0.0468 (16)	0.0335 (12)	0.0390 (14)	−0.0056 (11)	0.0020 (11)	−0.0019 (10)
C3	0.0389 (14)	0.0360 (12)	0.0356 (13)	0.0004 (11)	0.0006 (11)	0.0053 (10)
C4	0.0317 (13)	0.0359 (12)	0.0304 (12)	0.0021 (10)	0.0005 (10)	0.0013 (9)
C5	0.0457 (15)	0.0315 (12)	0.0314 (13)	−0.0014 (11)	−0.0010 (10)	−0.0005 (9)
C6	0.0466 (15)	0.0374 (12)	0.0335 (13)	−0.0011 (12)	−0.0016 (12)	0.0068 (10)
C7	0.0290 (14)	0.0668 (17)	0.0366 (14)	0.0049 (13)	0.0043 (11)	0.0116 (12)
C8	0.063 (2)	0.071 (2)	0.063 (2)	−0.0182 (17)	0.0129 (16)	0.0054 (16)
C9	0.058 (2)	0.072 (2)	0.0507 (18)	−0.0093 (16)	0.0045 (15)	−0.0012 (15)
C10	0.0330 (14)	0.0646 (17)	0.0361 (14)	0.0076 (13)	0.0063 (11)	0.0110 (12)
C11	0.0509 (17)	0.0654 (18)	0.0377 (15)	0.0104 (14)	0.0065 (12)	0.0146 (13)
C12	0.0494 (17)	0.0729 (19)	0.0318 (14)	0.0101 (15)	0.0042 (12)	0.0094 (13)

### Geometric parameters (Å, °)

**Table:**

S1—O2	1.439 (2)	C3—C4	1.383 (3)
S1—O1	1.447 (2)	C3—H3	0.9300
S1—O3	1.460 (2)	C4—C5	1.385 (3)
S1—C4	1.760 (2)	C5—C6	1.379 (3)
O3—H3A	0.8200	C5—H5	0.9300
O4—H4A	0.8501	C6—H6	0.9300
O4—H4B	0.8501	C7—C10	1.467 (4)
N1—C1	1.360 (3)	C8—C9	1.525 (4)
N1—H1A	0.8600	C8—H8A	0.9700
N1—H1B	0.8600	C8—H8B	0.9700
N2—C7	1.317 (3)	C9—H9A	0.9700
N2—C9	1.455 (4)	C9—H9B	0.9700
N2—H1	0.8600	C10—C12	1.387 (4)
N3—C7	1.317 (3)	C10—C11	1.405 (4)
N3—C8	1.474 (4)	C11—C12^i^	1.368 (4)
C1—C6	1.393 (3)	C11—H11	0.9300
C1—C2	1.403 (3)	C12—C11^i^	1.368 (4)
C2—C3	1.377 (3)	C12—H12	0.9300
C2—H2	0.9300		
			
O2—S1—O1	114.61 (15)	C4—C5—H5	119.9
O2—S1—O3	111.99 (14)	C5—C6—C1	121.2 (2)
O1—S1—O3	109.17 (13)	C5—C6—H6	119.4
O2—S1—C4	106.51 (12)	C1—C6—H6	119.4
O1—S1—C4	107.34 (13)	N3—C7—N2	111.6 (3)
O3—S1—C4	106.79 (11)	N3—C7—C10	124.3 (2)
S1—O3—H3A	109.5	N2—C7—C10	124.1 (2)
H4A—O4—H4B	120.0	N3—C8—C9	102.8 (3)
C1—N1—H1A	120.0	N3—C8—H8A	111.2
C1—N1—H1B	120.0	C9—C8—H8A	111.2
H1A—N1—H1B	120.0	N3—C8—H8B	111.2
C7—N2—C9	111.7 (2)	C9—C8—H8B	111.2
C7—N2—H1	124.2	H8A—C8—H8B	109.1
C9—N2—H1	124.2	N2—C9—C8	102.3 (2)
C7—N3—C8	110.3 (2)	N2—C9—H9A	111.3
N1—C1—C6	121.0 (2)	C8—C9—H9A	111.3
N1—C1—C2	121.2 (2)	N2—C9—H9B	111.3
C6—C1—C2	117.7 (2)	C8—C9—H9B	111.3
C3—C2—C1	121.0 (2)	H9A—C9—H9B	109.2
C3—C2—H2	119.5	C12—C10—C11	119.0 (3)
C1—C2—H2	119.5	C12—C10—C7	120.4 (2)
C2—C3—C4	120.3 (2)	C11—C10—C7	120.6 (2)
C2—C3—H3	119.8	C12^i^—C11—C10	120.3 (3)
C4—C3—H3	119.8	C12^i^—C11—H11	119.9
C3—C4—C5	119.5 (2)	C10—C11—H11	119.9
C3—C4—S1	120.81 (18)	C11^i^—C12—C10	120.7 (3)
C5—C4—S1	119.67 (18)	C11^i^—C12—H12	119.7
C6—C5—C4	120.2 (2)	C10—C12—H12	119.7
C6—C5—H5	119.9		

Symmetry codes: (i) −*x*+1, −*y*+2, −*z*+1.

### Hydrogen-bond geometry (Å, °)

**Table:**

*D*—H···*A*	*D*—H	H···*A*	*D*···*A*	*D*—H···*A*
O3—H3A···O4	0.82	2.00	2.809 (3)	168
N1—H1A···O2^ii^	0.86	2.22	2.960 (3)	145
N1—H1B···O3^iii^	0.86	2.43	3.200 (3)	150
N1—H1B···O1^iii^	0.86	2.46	3.170 (4)	141
N2—H1···O3^iv^	0.86	2.07	2.897 (3)	161
O4—H4A···N3^v^	0.85	2.08	2.760 (3)	136
O4—H4B···O1^vi^	0.85	2.20	2.817 (3)	130

Symmetry codes: (ii) *x*, −*y*+3/2, *z*+1/2; (iii) −*x*+1/2, −*y*+2, *z*+1/2; (iv) *x*+1/2, *y*, −*z*+1/2; (v) *x*−1/2, −*y*+3/2, −*z*+1; (vi) *x*−1/2, *y*, −*z*+1/2.
